# Extensive Genetic Diversity, Unique Population Structure and Evidence of Genetic Exchange in the Sexually Transmitted Parasite *Trichomonas vaginalis*

**DOI:** 10.1371/journal.pntd.0001573

**Published:** 2012-03-27

**Authors:** Melissa D. Conrad, Andrew W. Gorman, Julia A. Schillinger, Pier Luigi Fiori, Rossana Arroyo, Nancy Malla, Mohan Lal Dubey, Jorge Gonzalez, Susan Blank, William E. Secor, Jane M. Carlton

**Affiliations:** 1 Department of Biology, Center for Genomics and Systems Biology, New York University, New York, New York, United States of America; 2 New York City Department of Health and Mental Hygiene, Bureau of Sexually Transmitted Diseases Control, New York, New York, United States of America; 3 Division of Sexually Transmitted Disease Prevention, United States Centers for Disease Control and Prevention, Atlanta, Georgia, United States of America; 4 Division of Microbiology, Department of Biomedical Sciences, University of Sassari, Sassari, Italy; 5 Departamento de Infectómica y Patogénesis Molecular, Centro de Investigación y de Estudios Avanzados del Instituto Politécnico Nacional (CINVESTAV-IPN), Mexico City, Mexico; 6 Department of Parasitology, Postgraduate Institute of Medical Education and Research, Chandigarh, India; 7 Molecular Parasitology Unit, Faculty of Health Sciences, University of Antofagasta, Antofagasta, Chile; 8 Division of Parasitic Diseases and Malaria, Centers for Disease Control and Prevention, Atlanta, Georgia, United States of America; University of Pittsburgh, United States of America

## Abstract

**Background:**

*Trichomonas vaginalis* is the causative agent of human trichomoniasis, the most common non-viral sexually transmitted infection world-wide. Despite its prevalence, little is known about the genetic diversity and population structure of this haploid parasite due to the lack of appropriate tools. The development of a panel of microsatellite makers and SNPs from mining the parasite's genome sequence has paved the way to a global analysis of the genetic structure of the pathogen and association with clinical phenotypes.

**Methodology/Principal Findings:**

Here we utilize a panel of *T. vaginalis*-specific genetic markers to genotype 235 isolates from Mexico, Chile, India, Australia, Papua New Guinea, Italy, Africa and the United States, including 19 clinical isolates recently collected from 270 women attending New York City sexually transmitted disease clinics. Using population genetic analysis, we show that *T. vaginalis* is a genetically diverse parasite with a unique population structure consisting of two types present in equal proportions world-wide. Parasites belonging to the two types (type 1 and type 2) differ significantly in the rate at which they harbor the *T. vaginalis* virus, a dsRNA virus implicated in parasite pathogenesis, and in their sensitivity to the widely-used drug, metronidazole. We also uncover evidence of genetic exchange, indicating a sexual life-cycle of the parasite despite an absence of morphologically-distinct sexual stages.

**Conclusions/Significance:**

Our study represents the first robust and comprehensive evaluation of global *T. vaginalis* genetic diversity and population structure. Our identification of a unique two-type structure, and the clinically relevant phenotypes associated with them, provides a new dimension for understanding *T. vaginalis* pathogenesis. In addition, our demonstration of the possibility of genetic exchange in the parasite has important implications for genetic research and control of the disease.

## Author Summary

The human parasite *Trichomonas vaginali*s causes trichomoniasis, the world's most common non-viral sexually transmitted infection. Research on *T. vaginalis* genetic diversity has been limited by a lack of appropriate genotyping tools. To address this problem, we recently published a panel of *T. vaginalis*-specific genetic markers; here we use these markers to genotype isolates collected from ten regions around the globe. We detect high levels of genetic diversity, infer a two-type population structure, identify clinically relevant differences between the two types, and uncover evidence of genetic exchange in what was believed to be a clonal organism. Together, these results greatly improve our understanding of the population genetics of *T. vaginalis* and provide insights into the possibility of genetic exchange in the parasite, with implications for the epidemiology and control of the disease. By taking into account the existence of different types and their unique characteristics, we can improve understanding of the wide range of symptoms that patients manifest and better implement appropriate drug treatment. In addition, by recognizing the possibility of genetic exchange, we are more equipped to address the growing concern of drug resistance and the mechanisms by which it may spread within parasite populations.

## Introduction

Trichomoniasis is the most common non-viral sexually transmitted infection (STI) world-wide. Of the estimated 174 million new infections each year [1] – making it more prevalent than gonorrhea and chlamydia combined – ∼7 million occur in the United States [2]. Historically, trichomoniasis has been considered a self-clearing female ‘nuisance’ disease [3], but recent studies indicate that without antimicrobial treatment women may maintain chronic infections indefinitely, while men usually resolve infection without treatment [4]–[6]. In women, symptoms include malodorous vaginal discharge, vulval irritation and inflammation, and punctate microhemorrhages on the cervix known as ‘strawberry cervix’ [7]. Males, though often asymptomatic, can present with urethritis, urethral discharge and dysuria [8]. Trichomoniasis has been associated with severe reproductive health sequelae in both sexes, including, pelvic inflammatory disease [9] and adverse pregnancy outcomes [10], [11] in women, and prostatitis, infertility, and an increased incidence of aggressive prostate cancers in men [8],[12]. Perhaps most importantly, trichomoniasis has also been implicated in increasing sexual transmission of HIV up to two-fold [13], [14]. Because of the high prevalence of trichomoniasis, this translates into a significant number of global HIV infections [15].

*Trichomonas vaginali*s, the causative agent of human trichomoniasis, is a highly motile, aerotolerant, haploid eukaryotic parasite that resides in the urogenital tract and has an apparently simple life-cycle consisting of a trophozoite stage that is transmitted from host to host through sexual intercourse. The parasite itself can harbor a linear, double-stranded RNA virus known as *T. vaginalis* virus (TVV), which has been reported in approximately 50% of isolates, and may have important implications in virulence [16]. Currently, only the 5-nitroimidazole family of drugs (specifically metronidazole and tinidazole) is approved for the treatment of trichomoniasis; however, drug resistance (documented since this family of drugs was first used to treat the infection [17]) is a major concern, with estimates of up to 10% of infections not responding to treatment in the United States [18].

Current knowledge of *T. vaginalis* population genetics has been limited by a lack of appropriate tools. Crude genotyping markers such as random amplified polymorphic DNA (RAPDs) and restriction fragment length polymorphisms (RFLPs), have indicated genetic variation among *T. vaginalis* isolates and have inconclusively detected evidence of population structure [19]–[25]. These methods, however, are highly sensitive to contaminating DNA or to slight variation in conditions, which may influence the interpretation of data collected with these techniques. To address these limitations in existing methods of genetic characterization, we recently developed the first panel of *T. vaginalis*-specific microsatellite and single nucleotide polymorphisms (SNPs) as robust genetic markers [26]. These markers were utilized to sample the diversity of seven laboratory strains of *T. vaginalis* collected during the past >55 years and propagated *in vitro*, in some instances for more than a year. As a result of this work, we identified a significant amount of diversity across most loci in the seven strains, although differences existed between the topology of trees inferred from different types of markers.

However, limitations of that study included (a) a focus on laboratory strains collected many years previously; (b) sampling of a limited number of geographical regions; and (c) the small number of strains characterized. To assay the global diversity and population structure of *T. vaginalis*, we present here analysis of 235 *T. vaginalis* isolates from ten world-wide regions in Mexico, Chile, India, Australia, Papua New Guinea, Italy, Africa and the United States, including 19 clinical isolates recently collected from 270 women attending New York City sexually transmitted disease (STD) clinics. We find high genetic diversity within the *T. vaginalis* parasite, and a two-type population structure that is distributed in near equal frequencies world-wide. In addition, we show that the two types differ in the frequency in which they harbor TVV and in their metronidazole sensitivity. Finally, we present evidence of recent intragenic recombination and speculate on the possibility of a sexual life-cycle of the parasite in the absence of obvious sexual stages. Our findings enhance the understanding of the population genetics and diversity of *T. vaginalis* and suggest the possibility of genetic exchange in the parasite, which has wide-ranging implications for the epidemiology and control of human trichomoniasis.

## Methods

### Parasite Culture and Metronidazole Testing

Global strains and isolates obtained from collaborators, including references that provide details of their collection, are given in **Table S1**. All parasites were cultured in modified Diamond's media [27], supplemented with 10% horse serum, penicillin and streptomycin (Invitrogen) and iron solution composed of ferrous ammonium sulfate and sulfosalicylic acid (Fisher Scientific). Minimum lethal concentrations (MLCs) for metronidazole were determined under aerobic conditions by the lab of origin according to previously published protocols [22], unless otherwise indicated. A subset of the samples, the Secor Lab isolates (**Table S1**) are isolates sent to the CDC for drug resistance testing from patients who had previously failed at least two courses of standard therapy.

### Collection of clinical isolates from NYC STD clinics

Vaginal swabs were collected from 270 women attending eight New York City Department of Health and Mental Hygiene STD clinics in four of the five New York boroughs: Clinic G (N = 29) in the Bronx, Clinic D (N = 37) and Clinic F (N = 29) in Queens, Clinic A (N = 28) and Clinic E (N = 37) in Brooklyn, Clinic B (N = 36), and Clinic C (N = 41), and Clinic H (N = 33) in Manhattan. Approval for specimen collection and study was granted by the institutional review boards of NYU School of Medicine, the Centers for Disease Control and Prevention, and the New York City Department of Health and Mental Hygiene. IRB approval did not require informed consent since the study was deemed exempt. All samples were anonymized. Vaginal swabs collected during a routine pelvic examination, which would otherwise have been discarded, were used to inoculate InPouch TV culture kits [28]. Pouches were retrieved from clinics within three days of inoculation. Specimens were assigned a study ID and, after linkage to a limited set of demographic and clinical data, specimens were stripped of patient identifying information. Cultures were incubated at 37°C and examined for the presence of *T. vaginalis* by microscopy each day for five to seven days post-inoculation (PI) with vaginal swab. On day seven PI regardless of *T. vaginalis* diagnosis, 3 mL of culture was used for DNA extraction and the remaining culture media cryopreserved in 10% DMSO.

Diagnostic PCR was also used to diagnose *T. vaginalis*, using published methods. Two primer sets TVK3/TVK7 [29], [30] (5′-ATTGTCGAACATTGGTCTTACCCTC-3′)/(5′-TCTGTGCCGTCTTCAAGTATGC-3′) and TV16f-2/TV16r-2 [31] (5′-TGAATCAACACGGGGAAAC-3′)/(5′-ACCCTCTAAGGCTCGCAGT-3′) were used. Discrepancies between primer sets were resolved using a third primer set, BTub3f/BTUB_Bkmt [31] (5′-TCCAAAGGTTTCCGATACAGT-3′)/(5′-GTTGTGCCGGACATAATCATG-3′). All diagnostic PCRs were performed at least twice, and samples were considered positive if *T. vaginalis* was detected by wet mount, *in vitro* culture and/or by PCR with two different primers.

### DNA Isolation

The UNSET buffer and phenol-chloroform extraction method was used for DNA isolation of all samples as described [26].

### Microsatellite Genotyping

Isolates were genotyped at 21 *T. vaginalis*-specific microsatellite loci in 10 µL volumes as described [26]. Reactions were performed in duplicate and discrepancies were verified with a third reaction. *GeneMapper 4.0* (ABI, Foster City, CA) was used to score MS allele sizes. All calls were manually edited to discard data from poorly amplified reactions and to ensure that proper allele calls were assigned. Mixed infections were detected by the presence of multiple alleles at two or more loci. Due to the amplification biases described by Havryliuk *et al.* (2008) [32] in validating the criteria for distinguishing between minor alleles and stutter peaks (*i.e.*, minor peaks classified as >33% of the size of the major allele [33], [34]), we relied on the reproducibility of minor alleles over three independent rounds of amplification and electrophoretic analysis of labeled PCR products, and the presence of multiple alleles at two or more loci, in order to detect mixed infections. Electrophoretic readouts were also compared between samples to determine stutter patterns, and ambiguous minor alleles were ignored.

To ensure that the association of type 1 with TVV infection was not caused by interaction with the MS locus specific primers and the TVV genome, we performed a BLAST search of all known TVV genomes, including species I–IV, using each forward and reverse primer as a query. We found no more than 75% identity to any single primer, indicating that the TVV genome differs enough from the MS loci examined to prevent any unintended complementation.

### Population Genetic and Evolutionary Analyses of Microsatellite Data

Microsatellite allelic richness was estimated using *ADZE* [35], which implements the rarefaction method for analyzing allelic diversity across populations while correcting for sample size difference. Allelic richness estimates were graphed for each sample size (g) to estimate the sample size necessary to ensure that the majority of non-rare alleles had been detected.

Isolates were grouped according to geographical origin (or status as a laboratory strain) using the following ten categories: Laboratory (N = 5), Western United States *i.e.* west of the Mississippi River (N = 31), Eastern United States *i.e.* east of the Mississippi River (N = 51), Mexico (N = 11), Chile (N = 14), Italy (N = 12), Southern Africa (N = 19), Australia (N = 14), Papua New Guinea (PNG) (N = 30), and India (N = 1). Genetic diversity was determined by calculating expected heterozygosity (H_E_) at each locus, using the formula H_E_ = [*n*/(*n*−1)][1−∑*^n^_i = 1_ p^2^*] where *p* is the frequency of the *i*th allele and *n* is the number of alleles sampled and confirmed with *Arlequin3.11* [36]. Allelic richness (a measure of the number of alleles independent of sample size) per locus and sample (*R_s_*) and over samples (*R_t_*) was estimated using Mousadik and Petit's (1996) [37] method in *FSTAT 2.9.3.2* [38]. *FSTAT* estimates the expected number of alleles in a sub-sample of 2n genes, given that 2N genes have been sampled (*N*≥*n*), where *n* is fixed as the smallest number of individuals typed for a locus in a sample. The estimation is performed using the formula *R_s_* = Σ*^n^_i = 1_*[1−[[(2*N*−*N_i_*)/2*n*]/(2*N*/2*n*)], where *N_i_* is the number of alleles of type *i* among the 2*N* genes. For *R*_t_, the same sub-sample size *n* is kept, but *N* becomes the overall sample number of individuals genotyped at the locus under consideration. This program was chosen because allele frequencies are weighted according to sample sizes, important in our study due to the variation in the number of samples from different geographical regions. *Arlequin3.5* [36] was used to test for Fst between geographical origins.

The Bayesian clustering program *STRUCTURE 2.2* was used to assign isolates to *K* populations according to allele frequencies at each locus [39]. The program was run 10 times each for six *K* values (K = 1–6) with a burn-in period of 5×10^5^ iterations followed by 10^5^ iterations. The number of populations was inferred by plotting the log probability of the data [Ln P(D)] for each K value, followed by clusteredness calculations. Clusteredness measures the average relatedness of the individual membership coefficients (*Q*) and estimates the extent to which individual infections belong to a single cluster, rather than to a combination of clusters [40]. Population differentiation was confirmed using *Arlequin 3.5*. Two-way hierarchical clustering and inference of a minimum spanning network (MSN) were performed to validate clustering assignments determined using *STRUCTURE 2.2*. Two-way hierarchical clustering was performed on MS data using *JMP Genomics 5.0* (SAS), and missing data points were assigned a unique number (999) to allow for the inclusion of all samples in the analysis. MSNs were inferred from individual MS haplotypes profiles using *Network 4.516* [41], software developed to reconstruct all possible least complex phylogenetic trees using a range of data types.

### Sequencing of Single-Copy Genes

Loci TVAG_005070 (DNA mismatch repair homolog, postmeiotic segregation increased-1, PMS1), TVAG_302400 (MutL homolog 1a, Mlh1a), and TVAG_021420 (coronin, CRN) were PCR amplified, purified, and sequenced as described [26]. Nucleotide sequence data is available in the EMBL, GenBanks and DDBJ data bases under the accession numbers: JN380351–JN380802. Sequences were aligned to the reference sequence in GenBank and the alignments manually edited using *Sequencher* 4.8 (Gene Codes Corporation, Ann Arbor, MI). SNPs were manually verified and included any single nucleotide change that occurred in any single strain. All three genes were successfully sequenced in 94 isolates.

### Phylogenetic Analyses

*ModelGenerator* v. 0.85 [42] was used to infer phylogenies from single copy gene sequences, with the number of gamma categories set at 10 to identify appropriate nucleotide substitution models for each of the loci. *PhyML* [43] as part of *SeaView* v. 4.2.4 [44] was used to infer maximum likelihood (ML) phylogenies reconstructed by applying simultaneous NNI (Nearest Neighbor Interchange) and SPR (Subtree Pruning and Regrafting) moves on five independent random starting trees. Substitution rate categories were set at ten and transition/transversion (Ts/Tv) ratios, invariable sites and across-site rate variation were selected as indicated by *ModelGenerator*. Support values for the tree were obtained by bootstrapping 1000 replicates. We inferred the evolutionary relationship of type 1 and type 2 isolates by phylogenetic analyses of the concatenated protein sequences of the three single copy genes and their orthologs in *Tritrichomonas foetus* and *Pentatrichomonas hominis*. Sequences for *T. foetus* and *P. hominis* orthologs were obtained from mining low coverage Roche 454 sequence data of each species, and contigs with high sequence similarity were aligned and manually edited using *SeaView* v. 4.2.4 [44]. Indel regions were deleted from the alignment, leaving 1147 aa aligned sequence. BioNJ [45], a distance based phylogeny reconstruction method packaged in *SeaView* v. 4.2.4 was used to infer the phylogeny, using Poisson protein-level distances and 1000 bootstrap replicates.

### Linkage disequilibrium mapping and detection

To detect linkage disequilibrium (LD) between the MS loci we calculated pairwise LD using the exact test for haplotypic data encoded in *Arlequin*. For single copy gene loci, we utilized the 49 SNPs found in alleles of the 94 isolates, and used the *LDheatmaps* [46] package in *R* [47] to plot the standardized measure of linkage disequilibrium between pairs of sites, r^2^. As this program is designed for diploid organisms, we modified our haploid data by making all SNP genotypes homozygous. *LIAN* software version 3.5 was used to calculate *I^S^_A_*, a standardized index of association that tests for multilocus linkage disequilibrium, for MS loci. *I^S^_A_* is defined as *I^S^_A_* = (V_D_/V_E_−1)(r−1), where *(V_D_)* is the variance of the number of alleles shared between all pairs of haplotypes observed in a population *(D)*, *(V_E_)* is the variance expected under random association of alleles, and r is the number of loci analyzed. *V_E_* is derived from 10,000 simulated data sets in which alleles were randomly reshuffled among haplotypes. For single copy gene loci, we used the software package *MultiLocus 1.3b* [48]. This program can accommodate haploid sequencing data and implements an algorithm for *I^S^_A_* that is independent of the number of loci analyzed.

### TVV Detection

TVV infection in each parasite isolate was determined by isolating total RNA from 8–10 mls of late log phase cultures. RNA isolation was performed using Trizol (Invitrogen) according to the manufacturer's instructions. A total of 1 µg of total RNA was electrophoresed on a 1% agarose gel; the presence of rRNA bands on the gel served as a loading control to ensure that RNA from approximately equal number of parasites was examined. Gels were stained with ethidium bromide and isolates were considered positive for TVV if the characteristic ∼4.5 kb dsRNA genome band was detected.

## Results

### Prevalence and Genetic Diversity of *T. vaginalis* in NYC STD Clinics

In order to sample the genetic diversity and deduce the population structure of extant *T. vaginalis* in the local population, we collected a total of 270 vaginal swabs from female patients undergoing a pelvic examination at eight STD clinics in four boroughs of New York City (NYC) during the Summer of 2008 (**Table 1**). The average patient age was 27.7 years, and the majority self-identified as black non-Hispanic (N = 133, 49%) or Hispanic (N = 83, 31%), with the remainder reporting as white non-Hispanic (N = 17, 6%), Asian non-Hispanic (N = 9, 3%), American Indian (N = 1, 0.4%), Multi-ethnic (N = 3, 1%), or other (N = 10, 4%). Data on ethnicity was unavailable for 14 patients (5%). Wet mount diagnosis was performed in all clinics whenever a laboratory technician was available. Wet mount detected five *T. vaginalis* infections, while *in vitro* culture using InPouch TV packs diagnosed 19 *T. vaginalis* infections, and PCR amplification using three different sets of diagnostic primers detected a total of 26 infections. All culture-positive infections were detected by PCR as well, and all wet mount-positive infections were detected by both culture and PCR diagnosis. Thus wet mount, when performed, detected a mere 36% of the infections detected by PCR, and only 42% of infections detected by InPouch culture, suggesting that this method of diagnosis is highly insensitive and detection and treatment of *T. vaginalis* would be improved through the use of more sensitive point-of-care tests. We detected *T. vaginalis* infections in 10% of women attending NYC STD clinics, which is lower than the prevalence found in other STD clinics in the United States, but remains within the published range of 8–47% [8].

**Table 1:** Prevalence of *T. vaginalis* in women attending New York City STD clinics.

Clinic	Clinic Location	No. swabs	No. positive by wet mount n/N* (%)	No. positive by culture (%)	No. positive by PCR (%)
A	Brooklyn	28	1/25 (4)	2 (7.1)	2 (7.1)
B	Manhattan	36	1/20 (5)	4 (11.1)	5 (13.9)
C	Manhattan	41	1/27 (3.7)	2 (4.9)	3 (7.3)
D	Queens	37	0/15 (0)	0 (0)	1 (2.7)
E	Brooklyn	37	0/19 (0)	5 (13.5)	7 (18.9)
F	Queens	29	2/25 (8)	3 (10.3)	3 (10.3)
G	Bronx	29	NA	3 (10.3)	5 (17.2)
H	Manhattan	33	0/30 (0)	0 (0)	0 (0)
**Total**		**270**	**5/161 (3.1)**	**19 (7.0)**	**26 (9.6)**

The number of vaginal swabs collected at each clinic is shown, followed by the number of swabs found to be positive for *T. vaginalis* by wet mount, *in vitro* culture and PCR. NA: technician not available at clinic for wet-mount diagnosis during duration of sample collection.

*: n represents the number of samples diagnosed as positive via wet mount, and N represents the number of samples tested by wet mount. Type 1 parasites are more likely to be infected with the *T. vaginalis* virus TVV and are more susceptible to metronidazole, compared to type 2 parasites.

To gauge the extent of *T. vaginalis* genetic diversity within NYC, we used our panel of 21 polymorphic microsatellite (MS) markers [26] to genotype 19 isolates (seven infections detected by PCR could not be revived in culture to produce sufficient quantities of DNA for genotyping). One of the 19 isolates genotyped (NYCE32) had more than two alleles at four MS loci, indicating a mixed infection, and was excluded from further analyses. We found that each of the remaining 18 single infections had a unique haplotype, indicating high genetic diversity of the parasite even within the geographically limited area of NYC. This finding was also reflected in the moderately high average expected heterozygosity (H_E_ = 0.67) and allelic richness estimate for a population size of g = 4 (A = 3.24). An average of 4.29 distinct alleles were identified per locus (**Table 2**).

**Table 2:** Statistics of all *T. vaginalis* isolates genotyped at 21 microsatellite loci.

Region	No. samples	Mixed (%)	No. single infections	Avg. H_E_ *	Avg. no. alleles*	Avg. allelic richness* ‡
Laboratory strains	5	0	5	0.62	2.81	2.77
NYC STD clinics	19	1 (5.3)	18	0.67	4.29	3.24
Western USA	31	0	31	0.66	5.38	3.42
Eastern USA†	55	4 (7.3)	51	0.65	5.81	3.36
Mexico	11	0	11	0.58	3.4	2.85
Chile	17	3 (17.6)	14	0.67	4.1	3.25
Italy	15	3 (20.0)	12	0.59	3.5	2.89
Southern Africa	23	4 (17.4)	19	0.52	3.76	2.81
Australia	17	3 (17.6)	14	0.64	3.9	3.17
PNG	36	6 (16.7)	30	0.57	4.1	2.89
India§	11	0	11	0	1	1
**All regions combined** †	**211**	**23** **(10.9)**	**198**	**0.66**	**8.52**	**3.47**

Categorization in Eastern and Western United States regions was determined by geographical origin to the east or west of the Mississippi River. Region assignment for each isolate can be found in **Table S1**.

*: Mixed infections are excluded from these statistics.

**†:** NYC STD clinic samples are present in the table as a separate region and are also included in the Eastern United States region.

**‡:** Allelic richness is calculated for a minimum sample size of four diploid individuals.

**§:** All Indian isolates had the same genotype, most likely due to cross-contamination during *in vitro* culture of the isolates cultured in the same lab over many years. India is considered as a sample size of one in all subsequent analyses.

### Genetic Diversity Studies of Global Isolates

To determine if the moderately high genetic diversity exhibited by *T. vaginalis* isolates in NYC was unique to this geographic region, we extended our studies to include a set of 231 global samples, collected from nine countries: the United States, Mexico, Chile, Italy, South Africa, Mozambique, Australia, Papua New Guinea (PNG), and India, and five standard laboratory strains commonly used in research labs (**Table S1**). Each sample was genotyped in duplicate for all 21 MS markers, and 216 were successfully genotyped at ≥13 of the loci (**Table 2**). A total of 23 mixed infections (10.6%) was identified among the 216 isolates, 22 of which were double infections, while a Chilean isolate (ANT1) appeared to be a triple infection (two alleles identified at six loci and three alleles at a seventh locus). These isolates were excluded from further analysis. We found only four pairs of isolates from different geographical regions that shared haplotypes: three pairs were isolated from the Western United States (isolates 886 and 1135; 938 and 907; 1020 and 1025), and one was collected from both the Eastern and Western United States (isolates 1027 and 1162). In contrast, all eleven Indian isolates shared the same haplotype, unfortunately due to cross-contamination during their continuous culture in the same laboratory over many years. For this reason, we collapsed the same 11 genotypes to a single data point. A lack of shared haplotypes exhibited by our world-wide collection of *T. vaginalis* isolates is not due to incomplete sampling because graphing allelic richness estimates for each sample size revealed that the sampling had captured the majority of non-rare alleles (**Figure S1**).

A variety of population genetics statistics for the 183 clinical single infections and 5 laboratory strains indicate that the global genetic diversity of *T. vaginalis* is high and stable from region to region. The mean expected heterozygosity across all MS loci is 0.66±0.197, ranging from 0.04 (MS03) to 0.83 (MS17) respectively, and with an average of 8.52 alleles per locus (minimum 3.0 at MS03 and maximum 29 at MS17; **Table S2**). The expected heterozygosity is similarly high throughout all regions (**Table 2**), although statistically significant differences were apparent. For example, the *T. vaginalis* isolates from Chile, Western and Eastern United States and Australia are more diverse while the Southern Africa, Mexico and PNG isolates comprise a slightly less diverse group. We measured population differentiation between the geographical regions using F_ST_ measurements in *Arlequin*, and found that the Southern African and PNG parasite populations were significantly differentiated from the other global populations. The Mexican population differed from all other populations with the exception of the Italian population. The Chilean population differed from that of the Eastern United States, which was similar to the populations of both the Western United States and Australia (**Table S3**). Overall, we found that the least diverse groups differed the most from other global populations.

### *T. vaginalis* Has a Two-type Population Structure

**Figure 1:**
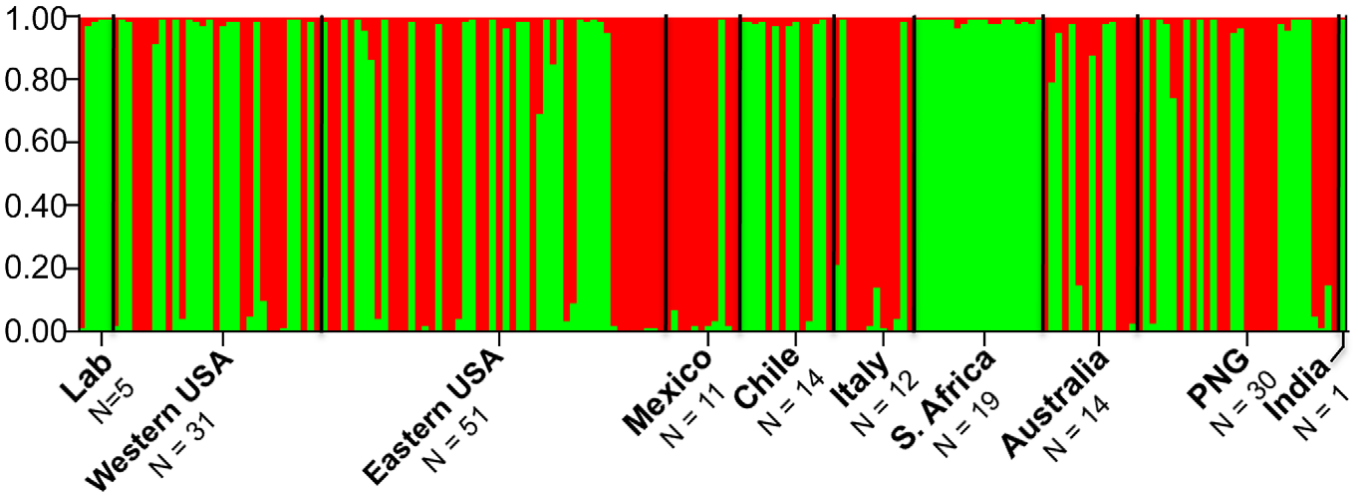
Two cluster population structure of *T. vaginalis*. A Bayesian clustering model implemented in STRUCTURE 2.2 indicates a two-cluster population structure for *T. vaginalis* (N = 188). The green cluster (type 1, (N = 95)) and red cluster (type 2, (N = 93) are found in each geographical location, with the exception of Southern Africa where all samples are type 1, and Mexico, which has a statistically significant over-representation of type 2. PNG: Papua New Guinea. Isolates included in each geographical location can be found in **Table S1**.

Next, we looked for population structure among the 188 global isolates (**Table S1**). Using the Bayesian clustering model implemented in *STRUCTURE 2.2* [39], the most probable number of clusters (populations) was determined by plotting the log probability of the data [Ln P(D)] for each k value followed by clusteredness scores. *K* = 2 coincided with a significant dip in the log probability of the data and received the highest clusteredness value (0.95 averaged across 10 independent simulations; **Figure 1**). Interestingly, the two clusters, which we refer to as ‘type 1’ and ‘type 2’, are present at nearly equal frequencies and are well distributed among all geographical locations as defined in **Table S1**. Two exceptions to this are isolates from Southern Africa and Mexico, which are significantly biased towards type 1 and type 2, respectively. Independent testing of this population structure was provided by two-way hierarchical clustering, which produced an identical clustering pattern, assigning the same isolates to the same clusters, and provided further evidence for a distinct two-type structure (**Figure S2**). Minimum spanning networks showed similar population differentiation, although we did not find perfect correlation (**Figure S3**). No evidence for further sub-population structure was found after repeating the analysis on each type individually.

**Figure 2:**
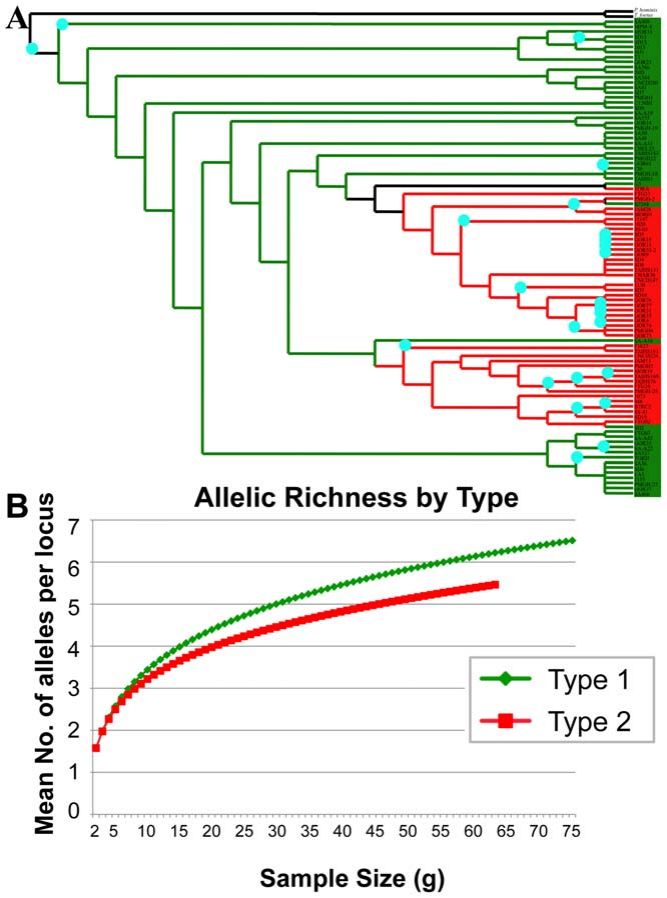
Type 1-like parasites appear to have given rise to type 2 parasites. (**A**) Phylogenetic tree of the evolutionary relationship of type 1 (green, N = 37) and type 2 (red, N = 57) single copy gene protein sequences, with *Pentatrichomonas hominis* and *Tritrichomonas foetus* protein sequences as outgroups. Protein sequences for CRN, PMS1 and Mlh1a were concatenated for each isolate and the tree was bootstrapped 1000 times. BioNJ methods were used for tree reconstruction. Blue dots indicate nodes where bootstrap support is ≥50. (**B**) The allelic richness by type was calculated with ADZE using microsatellite data for all isolates. Type 1 (green, N = 76) shows greater allelic richness than type 2 (red, N = 64), supporting the former as the ancestral clade and the latter as derived.

In addition to their geographical distribution, we investigated the temporal distribution of these two types. Likelihood ratios revealed no significant difference in the frequencies of the two types when isolates were categorized by the year in which they were isolated (**Figure S4**). To deduce which type is older in evolutionary history, we sequenced three single-copy genes – Coronin (CRN), MutL Homolog 1a (Mlh1a), and postmeiotic segregation increased 1 (PMS1), validated for phylogenetic analyses and described previously [26] – from 94 *T. vaginalis* isolates. Orthologs of these single-copy genes in two distant relatives of *T. vaginalis* [49], *Tritrichomonas foetus* (a trichomonad that infects the bovine urogenital tract) and *Pentatrichomonas hominis* (a human intestinal trichomonad), were identified, and used as outgroups to construct the phylogenetic tree for the concatenated protein sequences of the three genes. Although support at several nodes is weak, the phylogeny suggests that parasites more similar to type 1 existed before the emergence of parasites characteristic of type 2 (**Figure 2a**). We also found that type 1 has greater allelic richness than type 2, which further supports its ancestral nature, as the ancestral node would be expected to have accrued greater diversity (**Figure 2b**).

### Correlation of Phenotype to Genotype

We sought to identify phenotypic differences between the two types by correlating available clinical data with genotype (**Table 3**). Although the mean age of women infected with type 1 parasites is 35.0 years compared to 30.9 years for women infected with type 2 parasites, this difference was not statistically significant. We also found no statistically significant difference in the vaginal pH of women infected with the different types, nor did we find a significant difference in the percentage of isolates associated with a positive whiff test (a test used in the diagnosis of trichomoniasis). However, a highly significant difference was found in the minimum lethal concentration (MLC) of metronidazole necessary to kill isolates of the two types, with type 2 isolates demonstrating a mean MLC of 228.4 µg/ml of metronidazole, while type 1 isolates exhibited a mean MLC of 76.6 µg/ml of metronidazole. We also found that infection of *T. vaginalis* with the *T. vaginalis* virus (TVV) occurred significantly more frequently in type 1 isolates (112 of 154 isolates tested) than in type 2 (42 of 154 isolates; **Table 3**). Finally, our data – albeit insufficient for reliable statistics on this point – suggest that infections with type 1 parasites are more likely to be detected by wet-mount (microscopic) diagnosis than are infections with type 2 parasites; easier detection by microscopy might indicate a higher parasite load in type 1 infections.

**Table 3:** Correlations between *T. vaginalis* types and clinical phenotypes.

A. Discrete Correlate	Type 1No. positive/total number tested (%)	Type 2No. positive/total number tested (%)	Measure of statistical significance(name of test)
Whiff test positive	6/8 (75.0)	6/10 (60.0)	p = 0.50(Contingency Analysis)
Wet-mount positive	3/5 (60.0)	1/6 (16.7)	Insufficient data(Contingency Analysis)
TVV positive	54/74 (73.0)	2/79 (2.5)	p<0.0001*(Contingency Analysis)

B. Continuous Correlate	Type 1Mean, median (N)	Type 2Mean, median (N)	Measure of statistical significance(name of test)
Age	35.0, 36 (N = 17)	30.9, 28 (N = 24)	p = 0.21 (Wilcoxon)
Vaginal pH	5.06, 5.5 (N = 8)	4.6, 5 (N = 10)	p = 0.0959 (Wilcoxon)

**A.** The number of positive isolates out of the total number tested for each discrete correlate is shown, and partitioned according to whether those isolates are type 1 or type 2. **B.** The mean and median value for the total number (N) of isolates tested for several continuous correlates is shown, and partitioned according to whether those parasites are type 1 or type 2. All available clinical data were included in the analyses and specific analytical methods are indicated in parentheses.

*: indicates statistical significance.

### Genetic Exchange in *T. vaginalis*

We used several population genetics tools to address the question of whether genetic exchange occurs in *T. vaginalis*: (1) Pairwise linkage disequilibrium (LD: a locus to locus comparison to detect cases where specific alleles are found together more frequently than would be expected by chance alone); (2) the standardized index of association (I_A_^S^,: a measurement of the variance of the genetic distance between pairs of strains compared to variance in a shuffled matrix [50]); and (3) Maximum Chi-Squared Tests for recombination (Max Χ^2^: compares the distribution of polymorphic sites along paired sequences with those expected to occur by chance [51]). For parasites with a clonal population structure, *i.e.*, with no genetic exchange, the expectation would be to observe significant LD between loci and significant I_A_^S^ between MS and single-copy gene alleles.

**Figure 3:**
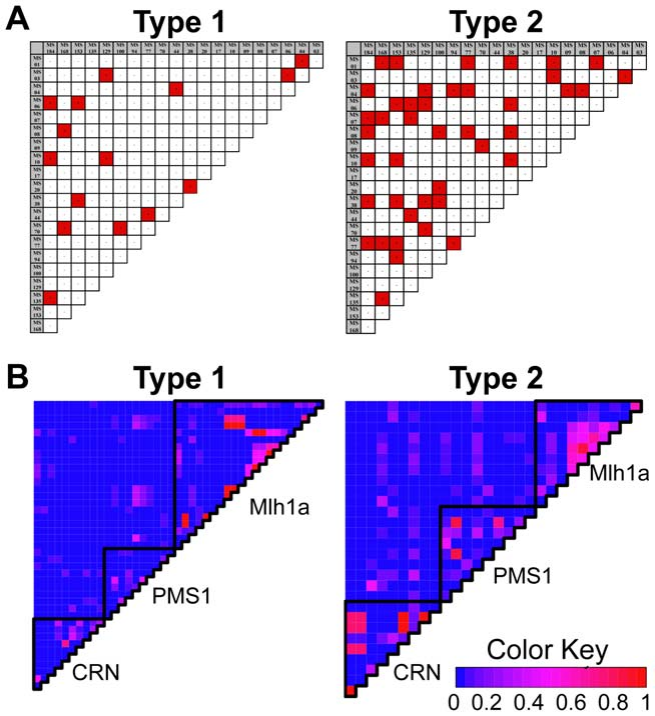
Linkage disequilibrium varies between type. (A) Pairwise linkage disequilibrium (LD) matrix for MS genotypes of type 1 (N = 95) and 2 (N = 93). Significant LD is highlighted in red. Type 2, with 42 cases of paired locus LD, shows a greater amount of LD in comparison to type 1 with 15 cases. (B) Heat maps indicating degree of LD between SNPs in single copy genes. Gene boundaries are indicated by black lines. The number of pairwise comparisons varies between the two types due to the difference in the number of polymorphisms analyzed, but a greater amount of pairwise LD in type 2 in comparison to type 1 is indicated.

We measured pairwise LD for each type using both MS loci and single-copy gene SNPs (**Figure 3**). Analysis of type 2 MS data revealed 42 cases of pairwise LD, while analysis of type 1 data MS revealed 15 (**Figure 3a**). This difference was confirmed upon calculation of I_A_^S^, which is highly significant in type 2 (I_A_^S^ = 0.0153, p≤1.00×10^5^) but not significant in type 1 (I_A_^S^ = 0.0006, p = 0.396), suggesting that the MS loci of isolates comprising the latter are in linkage equilibrium, while those in the former are not. We also measured LD within and between the single-copy genes and found minimal LD for both types (**Figure 3b**). Interestingly, strong LD is restricted and rare between the three genes in type 1, whereas type 2 is characterized by higher LD distributed among all three genes. However, in both cases, it appears that there is little genome-wide linkage, suggesting that the excess LD in type 2 may be due to either a recent bottleneck, a recent loss of recombination, or even to a recent expansion of evolutionarily favorable mutations within the population. These results are also consistent with the I_A_^S^ measurements calculated using *LDheatmap*. Breaking the three genes into linkage groups, we find that type 1 sequences have a non-significant I_A_^S^ (I_A_^S^ = 0.0296, p = 0.153), while type 2 is marginally non-significant (I_A_^S^ = 0.0598, p = 0.057), and becomes significant when the classical Index of Association (I_A_) is calculated (I_A_ = 0.1195, p = 0.046).

We tested for recombination events using Max Χ^2^ analysis in the bioinformatic program *START2* (**Table S4**). Among the 36 alleles found during sequencing of the single-copy gene CRN from 202 *T. vaginalis* isolates, we identified one putative recombination event between alleles CRN-25 and CRN-36 (Max Χ^2^ = 54.6422, p = 0.030). Using a p = 0.05 cutoff, we also identified 89 putative recombination events within 37 unique alleles identified from sequencing PMS1 of 144 *T. vaginalis* isolates, and 15 putative recombination events among the 41 unique alleles identified through sequencing Mlh1a of 110 *T. vaginalis* isolates.

**Figure 4:**
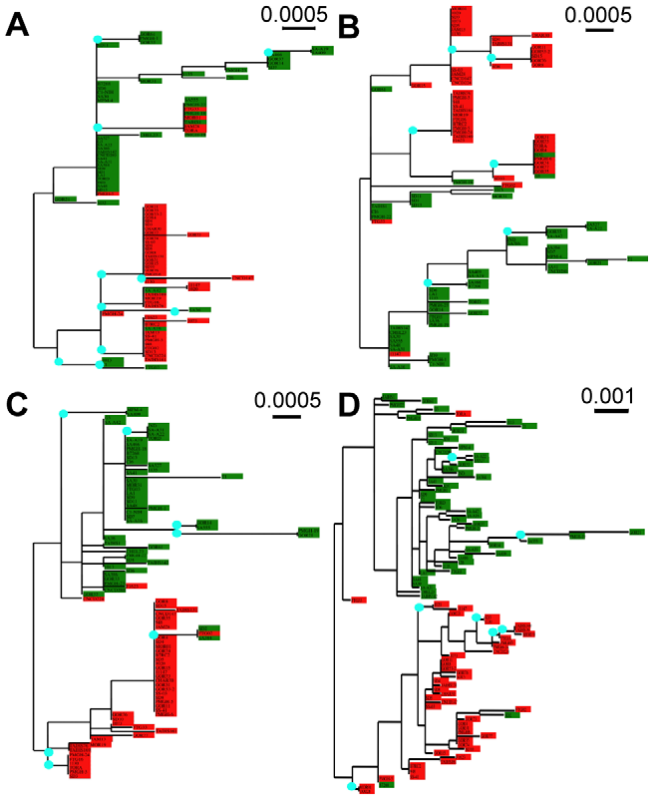
Phylogenies of single-copy genes. Phylogenetic trees were inferred from the DNA sequences for each of the three single copy genes (A) CRN (N = 94), (B) PMS1 (N = 94), and (C) Mlh1a (N = 94), and from the concatenation of all the three genes (N = 94; D). Blue dots indicate nodes where bootstrap support is ≥50. The clustering of type 1 (green) and type 2 (red) isolates supports the two-type population structure. Instances of unlike clustering resulting in non-homologous tree topology provide possible evidence for cases of recombination.

Finally, we compared the type assignments inferred by *STRUCTURE* from MS genotyping with phylogenies constructed from DNA sequences of each of the three single-copy genes (**Figure 4**). We found that the topologies are similar, each supporting a two-type population structure; however, in a number of cases, isolates from different types had identical SNP haplotypes within one gene but very different haplotypes within the other two genes. This suggests that some of our *T. vaginalis* isolates are recombinants that were generated through genetic exchange, which appears to occur within types and rarely between types. The DNA phylogenies are also shown in **Figure S5**, where isolates are color-coded by geographical origin.

## Discussion

The existence of genetically different *T. vaginalis* ‘types’ has been suggested by several previous studies. Stiles *et al.* (2000) found ten distinct HSP70 RFLP subtypes using 36 global reference strains and isolates collected from patients in Mississippi [19]. Rojas *et al.* (2004) utilized RAPD markers to genotype 40 isolates from Cuba and identified four subtypes with dendrograms inferred using UPGMA (unweighted pair group methods analysis) [23]. Meade *et al.* (2009) employed RFLPs to type 129 U.S. clinical isolates and used phylogenetic methods to identify two major groups composed of five subgroups [20]. Snipes *et al.* (2000) utilized RAPD polymorphisms and neighbor-joining phylogenetic methods with 63 U.S. strains and identified two groups [22]. Our own work with single-copy genes and microsatellites and a small number of laboratory strains also suggested a two-group genetic structure [26]. This current study, however, is the first to conclusively demonstrate the global distribution of two *T. vaginalis* types using robust, reproducible and diverse population genetic markers to genotype >230 isolates from nine regions around the globe. Our use of a set of powerful population genetic statistical tools, ranging from cluster analysis to two-way hierarchical clustering, along with tests for recombination, has allowed a rigorous investigation into *T. vaginalis* genetic diversity, population structure and genetic exchange. This information will be essential for future investigation into the parasite's biology.

We also demonstrate the utility of our panel of microsatellite markers to detect mixed genotype clinical infections. In a recent review, Balmer and Tanner discuss the theoretical and experimental work that suggests that mixed infections have a broad range of clinically relevant effects in a number of human pathogens, including effects on the host immune response, the ability to efficiently prevent and treat infection, and changes to pathogen and disease dynamics caused by intraspecific interactions, many of which can lead to pathogen evolution [52]. The availability of sensitive methods allowing the detection of multiple genotype infections in *T. vaginalis* research is likely to prove highly significant in understanding clinical trichomoniasis, as the ∼11% prevalence of mixed isolates identified in our study represents a non-trivial number of real *T. vaginalis* infections.

How did the striking two-type population structure of *T. vaginalis* arise? We propose three scenarios. We have previously hypothesized that the ancestor of *T. vaginalis* was an enteric pathogen (as are most trichomonads) that transitioned to the urogenital tract during its evolution [53]. It is possible that two separate colonization events occurred, producing two genetically distinct lineages within the urogenital niche. If genetic exchange in *T. vaginalis* is a rare event, this could explain the maintenance of the two lineages. Alternatively, it is possible that the two types evolved sympatrically after a single colonization event. The presence of the two types in nearly equal frequencies globally suggests that some form of balancing selection is maintaining both types in natural infections. Potential drivers for this balancing selection, *i.e.*, what causes one type to have an evolutionary advantage over the other under different selective conditions, may become apparent from studies characterizing phenotypic differences between the types. At this point, we have identified type-specific differences in frequency of *T. vaginalis virus* (TVV) infection and in susceptibility to metronidazole. As yet untested phenotypes that may be important in this context are: (a) differences in the ability to colonize the urogenital tracts of male *versus* female hosts; (b) a reduction in parasite fitness associated with metronidazole resistance when metronidazole treatment is not a selective force; or (c) differences in growth rates and virulence. Third and finally, the population structure reported here may have evolved when barriers arose that reduced the parasite's ability to undergo genetic exchange, causing gradual genetic isolation. In this respect it is interesting to note that ∼60% of the ∼160 Mb *T. vaginalis* genome consists of active transposable elements, virus-like repeats and retrotransposons [53], foreign DNA whose parasitism of the genome could have influenced the mechanics of genetic exchange, for example chromosome pairing. Indeed, transposable elements have been postulated to play a significant role in facilitating ectopic recombination in *Drosophila melanogaster* [54].

In contrast to the near-equal frequencies of the two *T. vaginalis* types detected in most regions, we found significant bias toward type 1 in Southern African samples and toward type 2 in Mexican samples (**Figure 1**). The low sample number (N = 11) for the Mexican isolates may explain why the frequencies appear to differ in this region; however, the 23 Southern African sources were comparatively diverse, comprised of asymptomatic women attending an antenatal clinic and symptomatic women attending an STD clinic.

Our finding of a highly significant difference in the frequency of TVV infection between type 1 and type 2 may have important implications for understanding variation in *T. vaginalis* virulence and disease pathogenesis. TVV has been implicated in affecting the expression of cysteine proteinases and of a highly immunogenic protein family (P250) on the parasite's surface [55]–[57]. In regard to the potential of such double-stranded RNA viruses to influence pathogenicity, it has been recently demonstrated that *Leishmania* RNA virus-1 controls the severity of mucocutaneous leishmaniasis by inducing Toll-like receptor 3, and ultimately inducing proinflammatory cytokines and chemokines that increases susceptibility to infection [58]. In addition, the greater prevalence of the virus in one type over the other may suggest differences in the functionality of the RNAi machinery that has been identified in the *T. vaginalis* genome [53].

The interesting observation that type 2 isolates have a significantly higher MLC for metronidazole may also have repercussions for understanding the mechanism(s) of metronidazole resistance in *T. vaginalis*, which has so far eluded scientists [59]–[61]. Isolates with an in vitro aerobic MLC of greater than or equal to 50 µg/ml are considered resistant to metronidazole [62], suggesting that the difference in median metronidazole MLCs of the two types may be clinically relevant (25 µg/ml vs. 200 µg/ml), and may have influenced the evolution of the species. For example, it is tempting to speculate that type 2 isolates may have diverged from type 1 isolates due to a selective advantage in being able to evade higher levels of metronidazole. This could account for the derived position of type 2 isolates in the *T. vaginalis* evolutionary tree (**Figure 2**), and could also explain their relative lack of diversity and genetic recombination, since there has been less time for mutations to accumulate in the more recently-evolved lineage. In addition, through limiting recombination, type 2 isolates may maintain favorable gene combinations such as those for increased metronidazole tolerance. It should be noted, however, that it is unlikely that metronidazole treatment has been adequately widespread to induce such selective evolution.

A major goal of this work was to use population genetics to identify evidence of genetic exchange in *T. vaginalis*. The parasite divides mitotically in the host, and no gamete form or cell fusion has been observed *in vitro*. However, circumstantial evidence suggests that *T. vaginalis* parasites may be capable of infrequent genetic recombination or may have only recently lost this ability. For example, analyses have revealed that closely related isolates share biologically relevant phenotypes, such as metronidazole resistance, but this pattern has no correlation with geographical origin, suggesting a spread of the phenotypes by recombination and the presence of strong selection [25]. In addition, Cui *et al.* (2010) found reassortment of polymorphic TMAC pseudogenes that cannot be explained by a strictly clonal population structure [63]. More persuasively, analyses of the *T. vaginalis* genome identified a complete set of conserved meiotic genes, suggesting that the meiotic process remains under, or has only recently been relieved of, conservative selection pressure [53], [64].

Tibayrenc and Ayala (2002) have outlined criteria and tests of clonality relating to eukaryotic parasites [65]. Among the criteria clonal organisms should meet are (1) the presence of over-represented, identical genotypes that are widespread; (2) evidence of linkage disequilibrium; and (3) the absence of segregating or recombinant genotypes. To address the first criterion, our studies found significant genotypic diversity (average H_E_ 0.66) and few shared haplotypes (total two in four isolates) among 188 *T. vaginalis* global isolates. The second criterion was addressed through analysis of the haplotypes generated using 21 microsatellite markers. Results of these tests indicated that *T. vaginalis* populations – and in particular type 1 – are in linkage equilibrium, indicative of genomes that have recently undergone recombination. Finally, we have identified recombination events between alleles of three different single-copy genes, providing evidence of recombinant genotypes. Taking these data as a whole, we infer that *T. vaginalis* does not fit the clonality model but rather appears to have undergone frequent genetic exchange in its recent evolutionary past. In addition, the presence of a complete set of meiosis-specific genes and the frequency (∼11%) at which mixed infections encounter each other in the host, suggest that the parasite continues to be capable of recombination, although the rate at which it occurs and under what conditions in natural populations remain to be determined.

The ability of *T. vaginalis* parasites to undergo genetic exchange has significant implications for the epidemiology and control of trichomoniasis. The Weismann hypothesis argues that genetic recombination functions to provide variation for natural selection to act upon, giving recombining species an evolutionary advantage in responding to selective pressures [66]. In other words, it provides opportunities for newly emerged, beneficial genes to be exchanged, potentially allowing them to be combined with other favorable genes, which may ultimately allow for the novel gene to become widespread throughout a population. This has obvious implications for such phenotypic traits as drug resistance, where a rare gene favorable to the parasite (and unfavorable to the host) may become widespread, with grave implications for treatment of the host. Not all consequences of genetic recombination are negative, however; should the mechanisms and conditions conducive to meiosis and genetic recombination in *T. vaginalis* be elucidated, important resources such as genetic crosses and quantitative trait loci (QTL) maps could be developed, significantly advancing our understanding of this neglected parasite.

## Supporting Information

Figure S1 **Allelic richness estimated by sample size for 147 clonal isolates with complete microsatellite genotype data.** After sampling ∼30 isolates, the rate of increase in sampling alleles begins to plateau, indicating that most non-rare alleles have been tested with our working sample size of 188 *T. vaginalis* isolates.
(DOC)

Figure S2 **Phylogenetic relationships of extant isolates.** Microsatellite genotypes were clustered using two-way hierarchical clustering, implemented in JMP Genomics 5.0. Each horizontal row indicates a single *T. vaginalis* isolate (N = 188), each vertical column represents a microsatellite locus (N = 21), and edges indicate genetic distances. Isolate names on the far left and edges are colored red (type 2 [N = 93]) or green (type 1, [N = 95]) based on the categorization indicated by STRUCTURE analysis. The dendrogram indicates a clear two-cluster structure. The geographical origin of each isolate is indicated by color-coding in the isolate origin column.
(TIF)

Figure S3 **Minimum spanning network relationships of extant isolates.** A minimum spanning network inferred by Network, utilizing microsatellite genotypes (N = 188). Edges and nodes (each indicating a single isolate) are color-coded to represent cluster assignment as indicated by STRUCTURE analysis.
(PPT)

Figure S4 **The frequencies of type 1 and type 2 do not change over time.** Isolates were categorized by their year of isolation (pre-1995, 1995–2000, 2001–2005, 2006–2010). The frequencies of type 1 and type 2 isolates were compared between these time periods using a likelihood ratio and Pearson test. The frequencies of the types do not differ significantly over time (likelihood, Χ^2^ = 4.627, p = 0.2012; Pearson, Χ^2^ = 4.585, p = 0.2048).
(DOCX)

Figure S5 **Phylogenies of single-copy genes.** Phylogenetic trees were inferred from the DNA sequences for each of the three single copy genes (A) CRN (N = 94), (B) PMS1 (N = 94), and (C) Mlh1a (N = 94), and from the concatenation of all the three genes (N = 94; D). This figure corresponds to Figure 4 from the text, with color-coding used to indicate geographical origin of each isolate.
(PPT)

Table S1 ***T. vaginalis*** **isolates included in the population genetics study.** When available, the list includes the name of each isolate, its specific geographical origin, the world region to which it is assigned for population studies, the provider of each strain, the year it was isolated from a patient, the TVV status determined by electrophoresis of total RNA extractions, the population type to which it clustered, whether the infected patient was recorded as being symptomatic or asymptomatic, the measured metronidazole MLC of the isolate, and references to publications in which the isolate has been included. NK: not known. *Antenatal samples from U.S. Navy wives or active duty U.S. Navy women. Since Navy personnnel are deployed all over the world, these isolates may not be typical of the Western USA.
(XLS)

Table S2 **Population genetic analysis of global isolates and lab strains.** Population genetic analysis of 188 global isolates and lab strains genotyped with 21 microsatellite loci. H_E_ = Expected Heterozygosity.
(DOC)

Table S3 **F_ST_ calculations for** ***T. vaginalis*** **isolates grouped according to their geographical origin.** F_ST_ calculations of 187 *T. vaginalis* isolates grouped according to their geographical origin. + indicates statistically significant F_ST_; − indicates no significant F_ST_. The Indian sample is excluded from the analysis because of its insufficient sample size.
(DOC)

Table S4 **SNP haplotypes for unique alleles of three single copy genes.** Haplotypes created from SNPs from unique alleles of the three single copy genes used in this study. (A) Coronin (CRN): 36 unique haplotypes identified from 202 T. vaginalis isolates (B) PMS1: 37 unique haplotypes identified from 144 T. vaginalis isolates; and (C) Mlh1a: 41 unique haplotypes identified from 110 T. vaginalis isolate. N: nonsynonymous SNP; S: synonymous SNP.
(XLS)
