# Supplementary figures and images for: Extensive Genetic Diversity, Unique Population Structure and Evidence of Genetic Exchange in the Sexually Transmitted Parasite Trichomonas vaginalis
Source: PLoS Negl Trop Dis. 2012 Mar 27;6(3):e1573. doi: 10.1371/journal.pntd.0001573 (PMC3313929; doi:10.1371/journal.pntd.0001573)

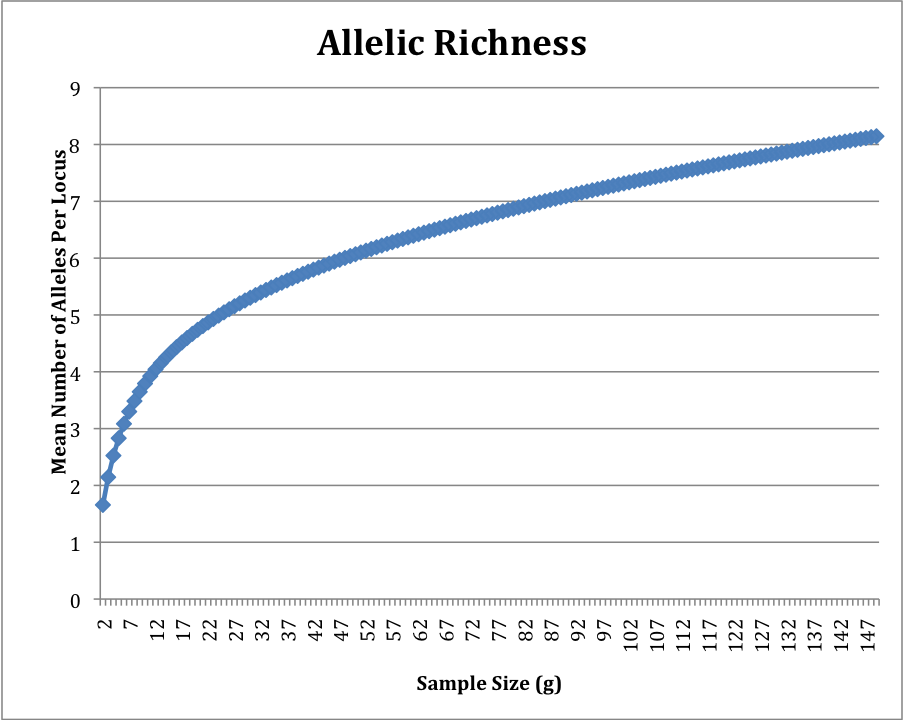

Supplement: Figure S1 — Allelic richness estimated by sample size for 147 clonal isolates with complete microsatellite genotype data. After sampling ∼30 isolates, the rate of increase in sampling alleles begins to plateau, indicating that most non-rare alleles have been tested with our working sample size of 188 T. vaginalis isolates. (DOC) [file pntd.0001573.s001.doc]

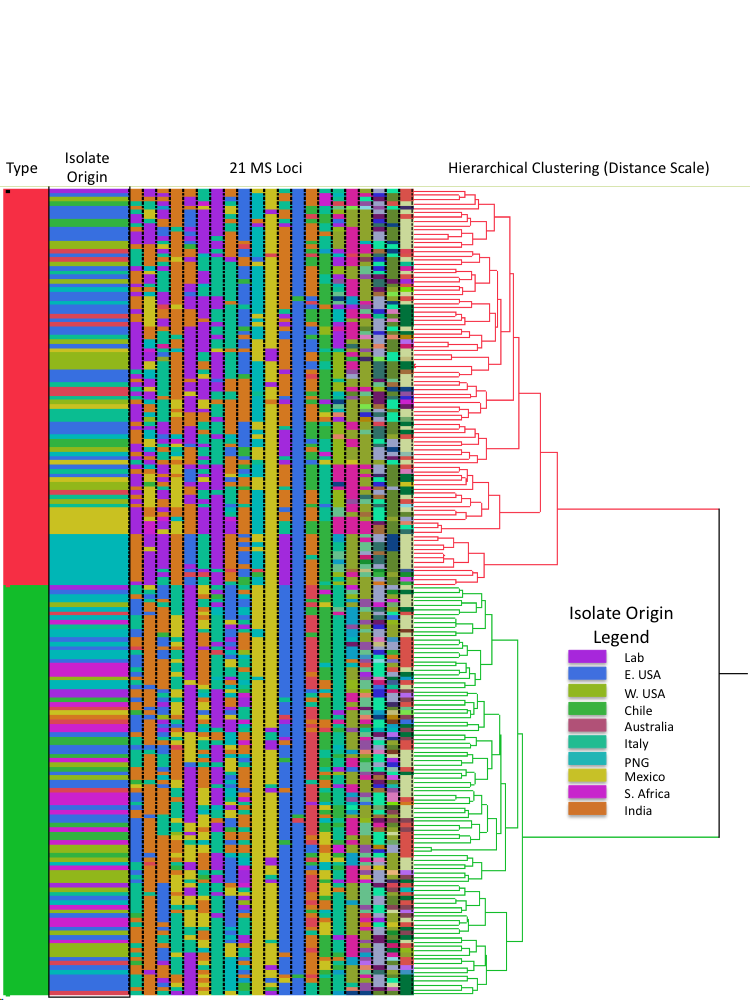

Supplement: Figure S2 — Phylogenetic relationships of extant isolates. Microsatellite genotypes were clustered using two-way hierarchical clustering, implemented in JMP Genomics 5.0. Each horizontal row indicates a single T. vaginalis isolate (N = 188), each vertical column represents a microsatellite locus (N = 21), and edges indicate genetic distances. Isolate names on the far left and edges are colored red (type 2 [N = 93]) or green (type 1, [N = 95]) based on the categorization indicated by STRUCTURE analysis. The dendrogram indicates a clear two-cluster structure. The geographical origin of each isolate is indicated by color-coding in the isolate origin column. (TIF) [file pntd.0001573.s002.tif]

## Slide 1
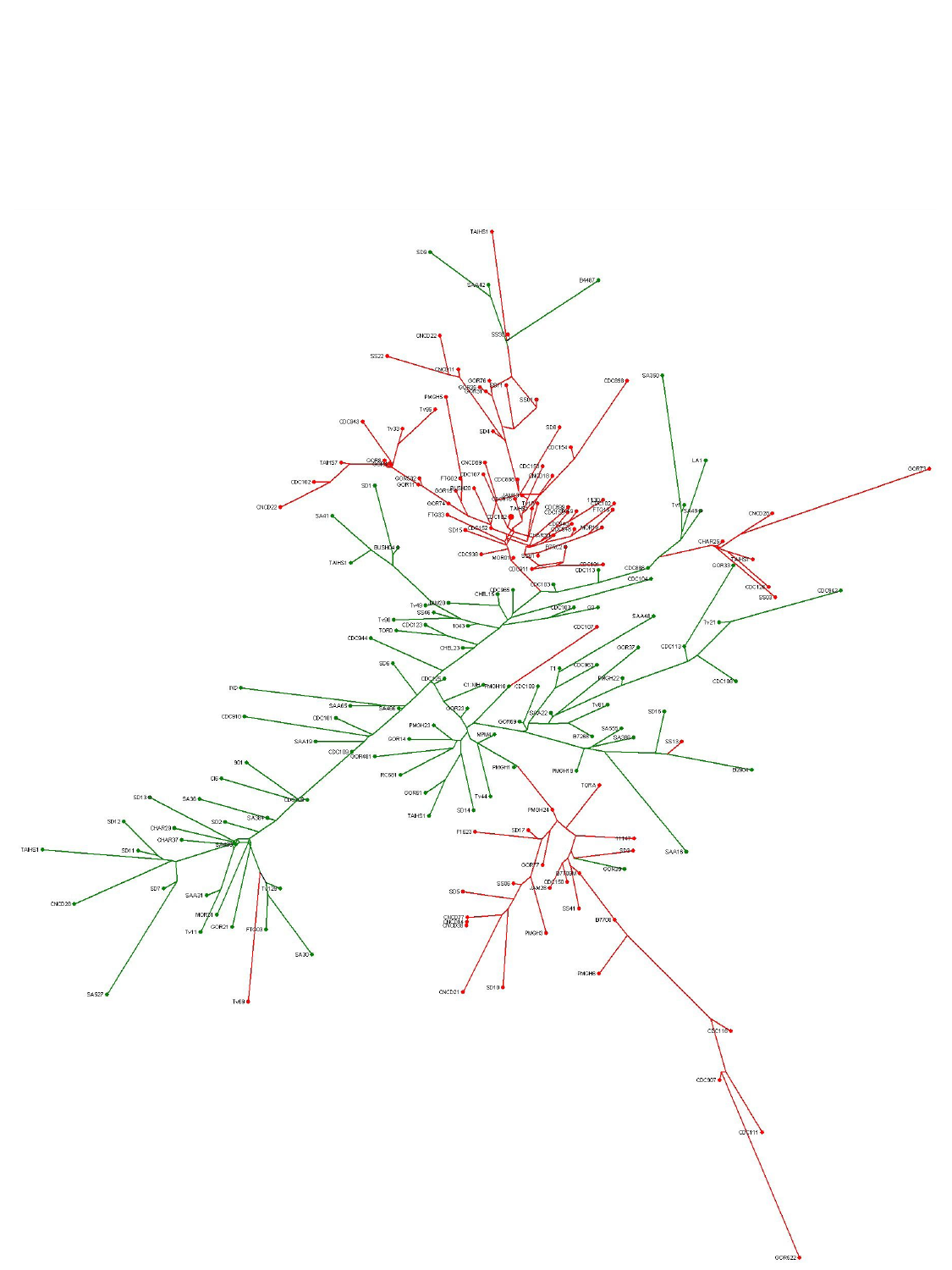

Supplement: Figure S3 — Minimum spanning network relationships of extant isolates. A minimum spanning network inferred by Network, utilizing microsatellite genotypes (N = 188). Edges and nodes (each indicating a single isolate) are color-coded to represent cluster assignment as indicated by STRUCTURE analysis. (PPT) [file pntd.0001573.s003.ppt]

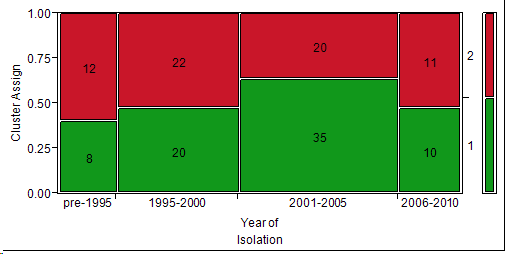

Supplement: Figure S4 — The frequencies of type 1 and type 2 do not change over time. Isolates were categorized by their year of isolation (pre-1995, 1995–2000, 2001–2005, 2006–2010). The frequencies of type 1 and type 2 isolates were compared between these time periods using a likelihood ratio and Pearson test. The frequencies of the types do not differ significantly over time (likelihood, Χ2 = 4.627, p = 0.2012; Pearson, Χ2 = 4.585, p = 0.2048). (DOCX) [file pntd.0001573.s004.docx]
