# Supplementary material for: Extensive Genetic Diversity, Unique Population Structure and Evidence of Genetic Exchange in the Sexually Transmitted Parasite Trichomonas vaginalis
Source: PLoS Negl Trop Dis. 2012 Mar 27;6(3):e1573. doi: 10.1371/journal.pntd.0001573 (PMC3313929; doi:10.1371/journal.pntd.0001573)
Supplement: Figure S5 — Phylogenies of single-copy genes. Phylogenetic trees were inferred from the DNA sequences for each of the three single copy genes (A) CRN (N = 94), (B) PMS1 (N = 94), and (C) Mlh1a (N = 94), and from the concatenation of all the three genes (N = 94; D). This figure corresponds to Figure 4 from the text, with color-coding used to indicate geographical origin of each isolate. (PPT) [file pntd.0001573.s005.ppt]

## Slide 1
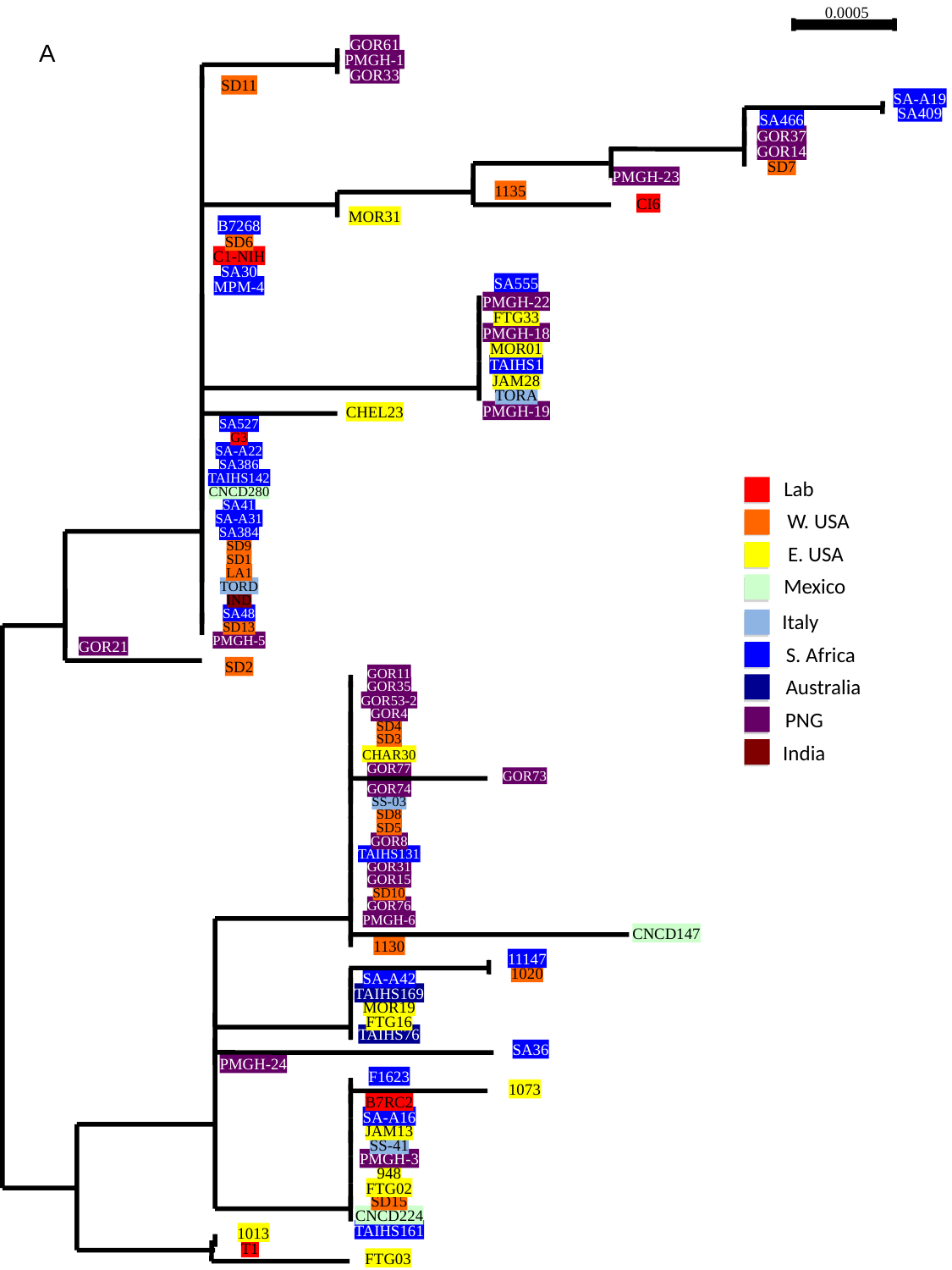

0.0005
A
GOR61
PMGH-1
GOR33
SD11
SA-A19
SA409
SA466
GOR37
GOR14
SD7
PMGH-23
1135
CI6
MOR31
B7268
SD6
C1-NIH
SA30
SA555
MPM-4
PMGH-22
FTG33
PMGH-18
MOR01
TAIHS1
JAM28
TORA
PMGH-19
CHEL23
SA527
G3
SA-A22
SA386
TAIHS142
Lab
W. USA
E. USA
Mexico
Italy
S. Africa
Australia
PNG
India
CNCD280
SA41
SA-A31
SA384
SD9
SD1
LA1
TORD
IND
SA48
SD13
PMGH-5
GOR21
SD2
GOR11
GOR35
GOR53-2
GOR4
SD4
SD3
CHAR30
GOR77
GOR73
GOR74
SS-03
SD8
SD5
GOR8
TAIHS131
GOR31
GOR15
SD10
GOR76
PMGH-6
CNCD147
1130
11147
1020
SA-A42
TAIHS169
MOR19
FTG16
TAIHS76
SA36
PMGH-24
F1623
1073
B7RC2
SA-A16
JAM13
SS-41
PMGH-3
948
FTG02
SD15
CNCD224
TAIHS161
1013
T1
FTG03

## Slide 2
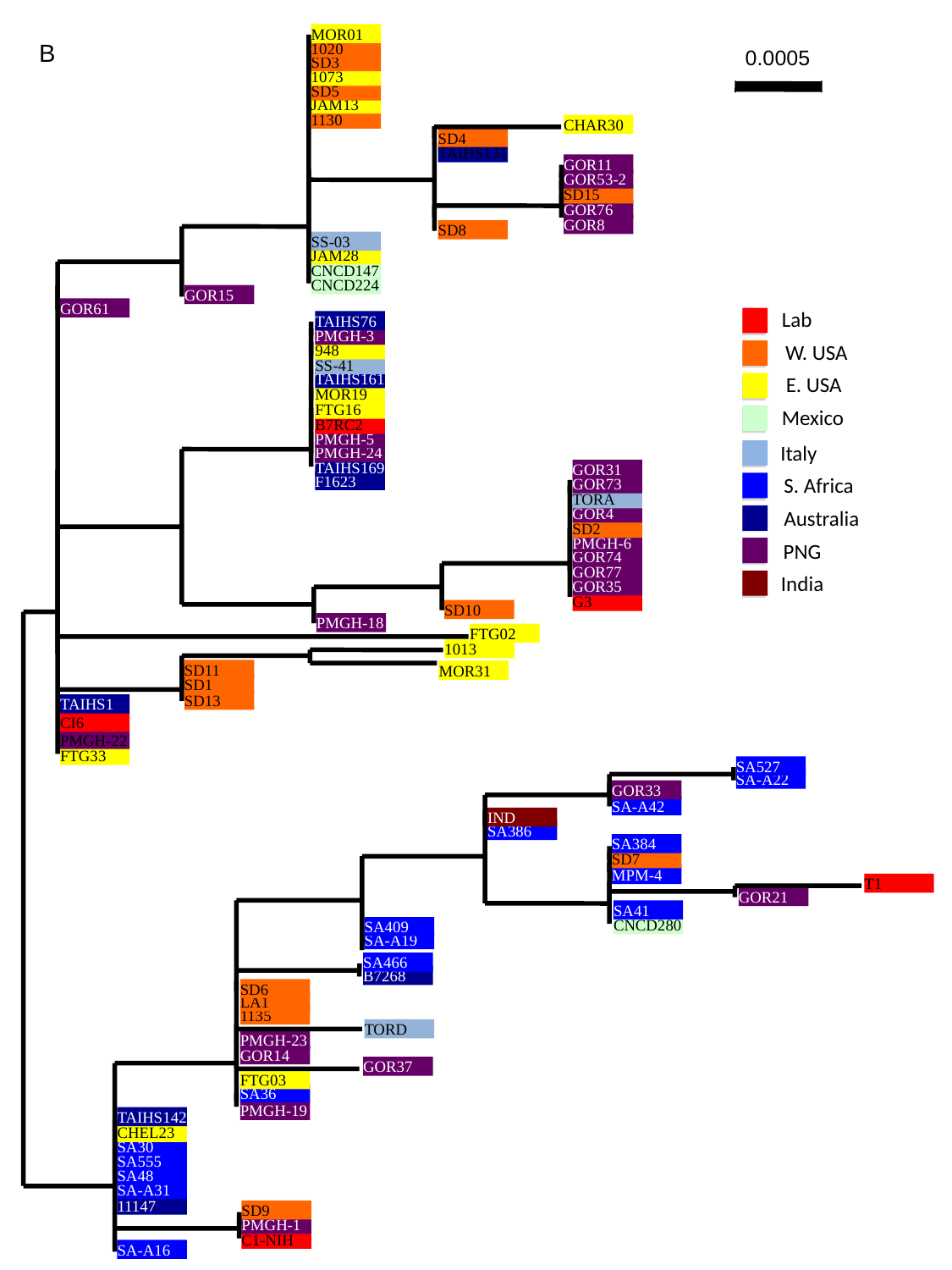

MOR01
B
1020
0.0005
SD3
1073
SD5
JAM13
1130
CHAR30
SD4
TAIHS131
GOR11
GOR53-2
SD15
GOR76
GOR8
SD8
SS-03
JAM28
CNCD147
CNCD224
GOR15
GOR61
Lab
W. USA
E. USA
Mexico
Italy
S. Africa
Australia
PNG
India
TAIHS76
PMGH-3
948
SS-41
TAIHS161
MOR19
FTG16
B7RC2
PMGH-5
PMGH-24
TAIHS169
GOR31
F1623
GOR73
TORA
GOR4
SD2
PMGH-6
GOR74
GOR77
GOR35
G3
SD10
PMGH-18
FTG02
1013
SD11
MOR31
SD1
SD13
TAIHS1
CI6
PMGH-22
FTG33
SA527
SA-A22
GOR33
SA-A42
IND
SA386
SA384
SD7
MPM-4
T1
GOR21
SA41
CNCD280
SA409
SA-A19
SA466
B7268
SD6
LA1
1135
TORD
PMGH-23
GOR14
GOR37
FTG03
SA36
PMGH-19
TAIHS142
CHEL23
SA30
SA555
SA48
SA-A31
11147
SD9
PMGH-1
C1-NIH
SA-A16

## Slide 3
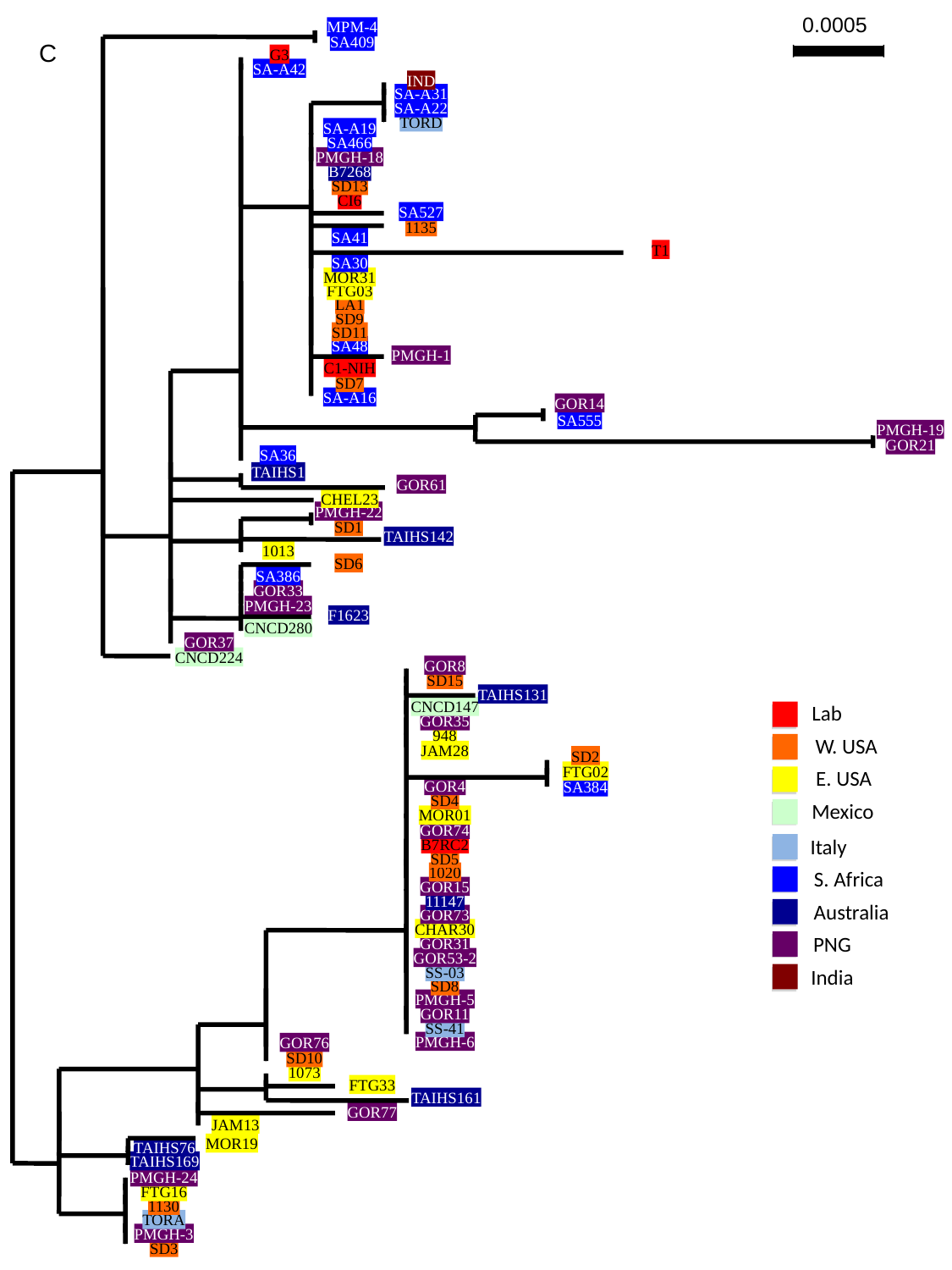

0.0005
MPM-4
C
SA409
G3
SA-A42
IND
SA-A31
SA-A22
TORD
SA-A19
SA466
PMGH-18
B7268
SD13
CI6
SA527
1135
SA41
T1
SA30
MOR31
FTG03
LA1
SD9
SD11
SA48
PMGH-1
C1-NIH
SD7
SA-A16
GOR14
SA555
PMGH-19
GOR21
SA36
TAIHS1
GOR61
CHEL23
PMGH-22
SD1
TAIHS142
1013
SD6
SA386
GOR33
PMGH-23
F1623
CNCD280
GOR37
CNCD224
GOR8
SD15
TAIHS131
CNCD147
Lab
W. USA
E. USA
Mexico
Italy
S. Africa
Australia
PNG
India
GOR35
948
JAM28
SD2
FTG02
GOR4
SA384
SD4
MOR01
GOR74
B7RC2
SD5
1020
GOR15
11147
GOR73
CHAR30
GOR31
GOR53-2
SS-03
SD8
PMGH-5
GOR11
SS-41
PMGH-6
GOR76
SD10
1073
FTG33
TAIHS161
GOR77
JAM13
MOR19
TAIHS76
TAIHS169
PMGH-24
FTG16
1130
TORA
PMGH-3
SD3

## Slide 4
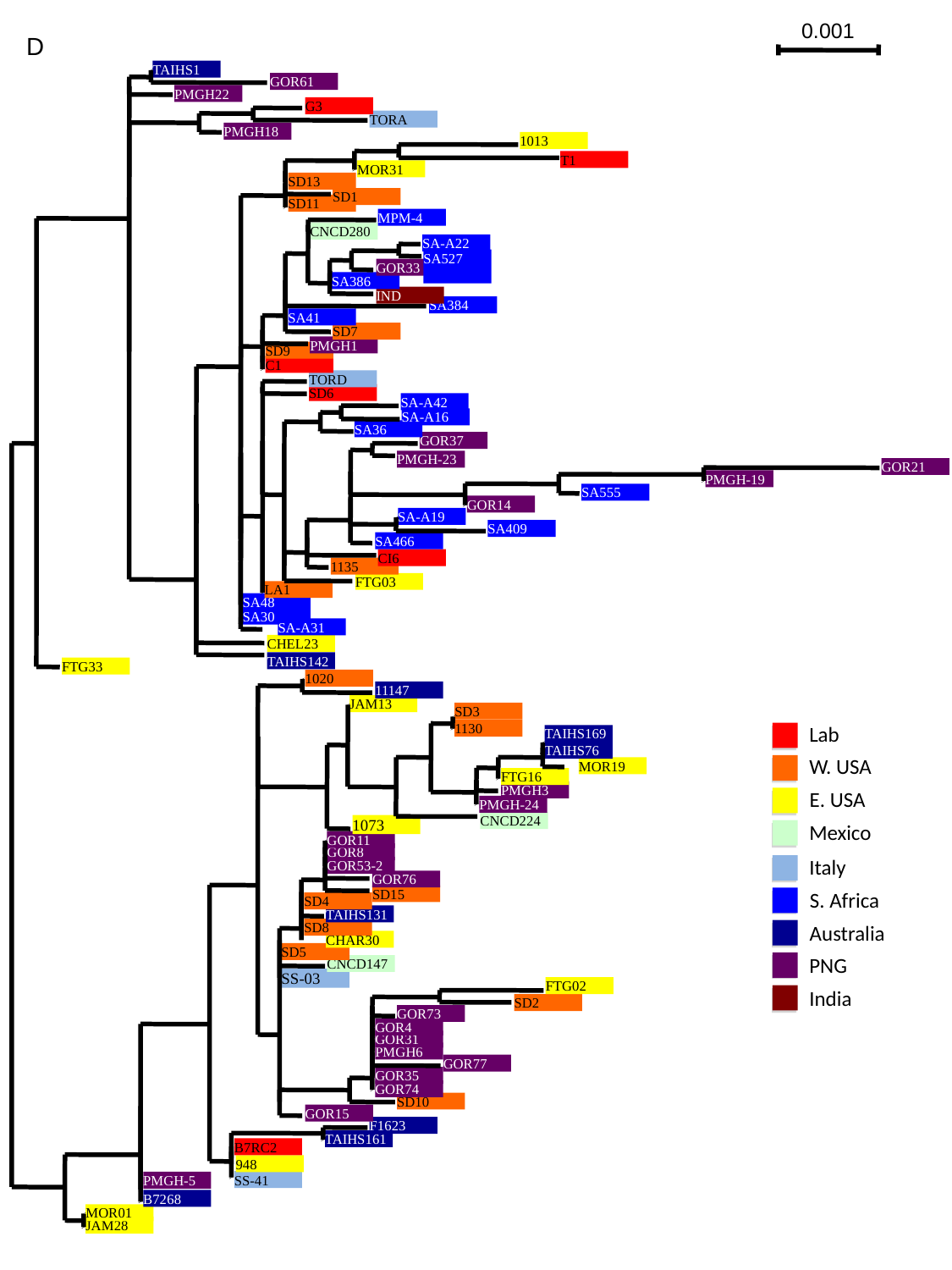

0.001
TAIHS1
GOR61
PMGH22
G3
TORA
PMGH18
1013
T1
MOR31
SD13
SD1
SD11
MPM-4
CNCD280
SA-A22
SA527
GOR33
SA386
IND
SA384
SA41
SD7
PMGH1
SD9
C1
TORD
SD6
SA-A42
SA-A16
SA36
GOR37
PMGH-23
GOR21
PMGH-19
SA555
GOR14
SA-A19
SA409
SA466
CI6
1135
FTG03
LA1
SA48
SA30
SA-A31
CHEL23
TAIHS142
FTG33
1020
11147
JAM13
SD3
1130
TAIHS169
TAIHS76
MOR19
FTG16
PMGH3
PMGH-24
CNCD224
1073
GOR11
GOR8
GOR53-2
GOR76
SD15
SD4
TAIHS131
SD8
CHAR30
SD5
CNCD147
SS-03
FTG02
SD2
GOR73
GOR4
GOR31
PMGH6
GOR77
GOR35
GOR74
SD10
GOR15
F1623
TAIHS161
B7RC2
948
SS-41
PMGH-5
B7268
MOR01
JAM28
D
Lab
W. USA
E. USA
Mexico
Italy
S. Africa
Australia
PNG
India
