# Supplementary material for: Extensive Genetic Diversity, Unique Population Structure and Evidence of Genetic Exchange in the Sexually Transmitted Parasite Trichomonas vaginalis
Source: PLoS Negl Trop Dis. 2012 Mar 27;6(3):e1573. doi: 10.1371/journal.pntd.0001573 (PMC3313929; doi:10.1371/journal.pntd.0001573)
Supplement: Table S2 — Population genetic analysis of global isolates and lab strains. Population genetic analysis of 188 global isolates and lab strains genotyped with 21 microsatellite loci. HE = Expected Heterozygosity. (DOC) [file pntd.0001573.s007.doc]

**Table S2.** Population genetic analysis of global isolates and lab strains.

| **Locus** | **No. samples genotyped** | **H_E_** | **No. alleles** | **Avg. allelic richness** |
| --- | --- | --- | --- | --- |
| MS01 | 187 | 0.69 | 9 | 3.74 |
| MS03 | 188 | 0.04 | 3 | 1.17 |
| MS04 | 176 | 0.68 | 12 | 3.40 |
| MS06 | 188 | 0.68 | 11 | 3.69 |
| MS07 | 184 | 0.66 | 6 | 3.06 |
| MS08 | 187 | 0.66 | 6 | 3.46 |
| MS09 | 185 | 0.49 | 5 | 2.15 |
| MS10 | 149 | 0.71 | 6 | 3.47 |
| MS17 | 168 | 0.83 | 29 | 5.09 |
| MS20 | 185 | 0.20 | 4 | 1.72 |
| MS38 | 187 | 0.79 | 16 | 4.68 |
| MS44 | 180 | 0.75 | 5 | 3.64 |
| MS70 | 163 | 0.79 | 8 | 4.35 |
| MS77 | 187 | 0.66 | 6 | 3.19 |
| MS94 | 188 | 0.76 | 7 | 3.75 |
| MS100 | 187 | 0.56 | 7 | 2.44 |
| MS129 | 159 | 0.73 | 8 | 3.72 |
| MS135 | 186 | 0.78 | 9 | 4.17 |
| MS153 | 187 | 0.78 | 8 | 3.95 |
| MS168 | 187 | 0.81 | 8 | 4.42 |
| MS184 | 188 | 0.73 | 6 | 3.6 |
| **Average** | **180.76** | **0.66 ± 0.2** | **8.52** | **3.47** |

Population genetic analysis of 188 global isolates and lab strains genotyped with 21 microsatellite loci. H_E_ = Expected Heterozygosity.
