# Supplementary material for: Extensive Genetic Diversity, Unique Population Structure and Evidence of Genetic Exchange in the Sexually Transmitted Parasite Trichomonas vaginalis
Source: PLoS Negl Trop Dis. 2012 Mar 27;6(3):e1573. doi: 10.1371/journal.pntd.0001573 (PMC3313929; doi:10.1371/journal.pntd.0001573)
Supplement: Table S3 — FST calculations for T. vaginalis isolates grouped according to their geographical origin. FST calculations of 187 T. vaginalis isolates grouped according to their geographical origin. + indicates statistically significant FST; − indicates no significant FST. The Indian sample is excluded from the analysis because of its insufficient sample size. (DOC) [file pntd.0001573.s008.doc]

**Table S3.** F_ST_ calculations for *T. vaginalis* isolates grouped according to their geographical origin.

F_ST_ calculations of 187 *T. vaginalis* isolates grouped according to their geographical origin. + indicates statistically significant F_ST_; – indicates no significant F_ST_. The Indian sample is excluded from the analysis because of its insufficient sample size.
